# Tadalafil Treatment Improves Inflammation, Cognitive Function, And Mismatch Negativity Of Patients With Low Urinary Tract Symptoms And Erectile Dysfunction

**DOI:** 10.1038/s41598-019-53136-y

**Published:** 2019-11-19

**Authors:** Amparo Urios, Felipe Ordoño, Raquel García-García, Alba Mangas-Losada, Paola Leone, Juan José Gallego, Andrea Cabrera-Pastor, Javier Megías, Juan Fermin Ordoño, Vicente Felipo, Carmina Montoliu

**Affiliations:** 1 Fundación Investigación Hospital Clínico Valencia, INCLIVA, Valencia, Spain; 2 Servicio Urología, Hospital Arnau Vilanova, Valencia, Spain; 3 Laboratorio de Neurobiologia, Centro Investigación Príncipe Felipe, Valencia, Spain; 4 Departamento Patología, Facultad Medicina, Universidad Valencia, Valencia, Spain; 5 Servicio Neurofisiología, Hospital Arnau Vilanova, Valencia, Spain, Psychopatology and Neurophysiology Unit, Paterna Mental Health Center, CIBERSAM, Valencia, Spain

**Keywords:** Medical research, Neurological manifestations

## Abstract

Patients with Benign prostatic hyperplasia, low urinary tract symptoms, and erectile dysfunction (BPH/LUTS-ED) present chronic inflammation. We studied in patients with BPH/LUTS-ED the effect of tadalafil treatment (5 mg/day) on changes in peripheral inflammation, cognitive function, and the auditory evoked potential, “mismatch negativity” (MMN). Nine patients with BPH/LUTS-ED and 12 controls performed psychometric tests, MMN. IL-6, IL-17, IL-18, cGMP and CD4^+^CD28^−^ autoreactive T-cells were measured in blood. Patients with BPH/LUTS-ED performed psychometric tests, MMN, and blood extraction at baseline and after tadalafil treatment. Patients with BPH/LUTS-ED showed increased CD4^+^CD28^−^ autoreactive T-cells (p < 0.05), and higher levels of pro-inflammatory interleukins IL-6 (p < 0.001), IL-17 and IL-18 (p < 0.05), compared to controls. Patients got lower scores than controls in psychometric tests assessing mental processing speed and attention (p < 0.05), and showed lower amplitude (p < 0.01) and area (p < 0.05) of MMN wave than controls. Inflammatory, psychometric and electrophysiological parameters were normalized after tadalafil treatment. In conclusion, there is a pro-inflammatory environment in blood in patients with BPH/LUTS-ED which would induce cognitive impairment and alter MMN. Phosphodiesterase-5 inhibition with tadalafil exerts anti-inflammatory effects and ameliorates cognitive function and MMN parameters. Tadalafil could be a promising candidate for chronic treatment in other inflammatory pathologies associated with mild cognitive impairment.

## Introduction

Benign prostatic hyperplasia (BPH) is one of the most common benign diseases in aging man which becomes a clinical entity when associated with lower urinary tract symptoms (LUTS). BPH/LUTS, or benign prostatic hyperplasia associated with lower urinary tract symptoms, can lead to benign prostatic hypertrophy, and is usually associated with erectile dysfunction (ED)^1^.

The etiology of BPH/LUTS is multifactorial and age and volume of the prostate are associated with its development. These factors can identify patients with increased risk of progression and it is advisable to initiate early treatment.

Standard treatments for BPH/LUTS include alpha-blockers and 5 alpha-reductase inhibitors, alone or in combination therapy. These drugs are effective, but may be associated with unwanted side effects, including dizziness, hypotension, and sexual dysfunction^2^.

Among the mechanisms involved in the pathology of BPH/LUTS, the Nitric oxide (NO)/cGMP pathway has an important functional role^3^, and all key enzymes of this pathway, nitric oxide synthase, PKG-1 and phosphodiesterase 5 (PDE5) are expressed in the prostatic tissue. NO exerts a general inhibitory effect of muscle tone on the lower urinary tract. A decrease in NO-mediated relaxation of smooth prostatic muscle could be involved in BPH/LUTS pathology. Increasing the NO/cGMP pathway activity by inhibiting PDE5 with inhibitors, such as tadalafil, is used in daily clinical practice for treatment of benign prostatic hyperplasia and erectile dysfunction^4^. Tadalafil is currently being used to treat pulmonary arterial hypertension, benign prostatic hyperplasia and erectile dysfunction^4,5^. PDE5 inhibitors have also recently emerged as a potential therapeutic strategy for neuroinflammatory, neurodegenerative, and memory loss diseases^6,7^.

In rat models of minimal hepatic encephalopathy impairment of learning is due to reduced cGMP in brain and can be reversed by increasing cGMP with PDE5 inhibitors^7^.

Chronic inflammation plays an important role in BPH/LUTS pathogenesis^8,9^ and contributes to the clinical progression of BPH-related syndromes. Most of the removed hyperplastic prostates contain inflammatory cells which produce a large number of cytokines that could play a role in the growth of prostate cells and hyperplastic change^10^. Chronic low-grade systemic inflammation may be involved in the etiology of BPH^11^. Tadalafil, but not sildenafil or vardenafil, reduces pro-inflammatory factors in endothelial cells in culture^12^ and in isolated pulmonary arteries of rats^13^.

Erectile dysfunction is commonly associated with depression, and treatment with PDE5 inhibitors improves cognitive functions and depression, in addition to erectile dysfunction^14^. Therefore, treatment of BPH/LUTS-ED with tadalafil could improve patients’ quality of life and their cognitive functions.

Reduced attention capacity affects cognitive function and the ability to perform daily living tasks. The area under the curve of the evoked potential “mismatch negativity” (MMN) is reduced in cirrhotic patients with minimal hepatic encephalopathy, and correlates with attention deficits^15^.

We hypothesized that in patients with BPH/LUTS-ED chronic inflammation would be associated with impaired cognitive function and MMN, and that treatment with tadalafil will improve inflammation and cognitive function, as sildenafil in animal models. Tadalafil should also improve the “mismatch negativity”.

The objectives of this work were to study in patients with BPH/LUTS-ED the effect of tadalafil treatment on changes in peripheral inflammation, cognitive function, and on the auditory evoked potential “mismatch negativity”.

## Results

**Figure 1:**
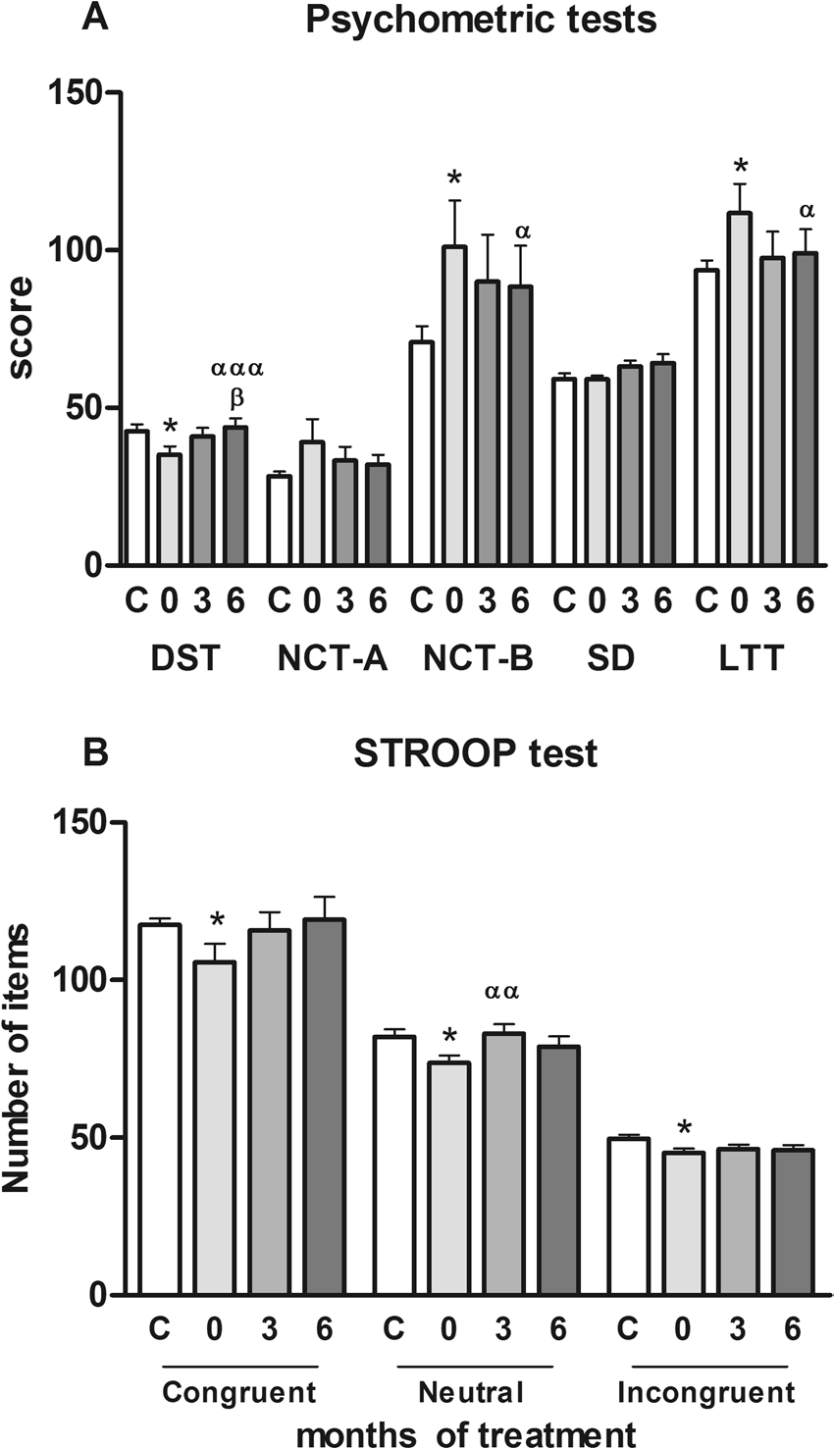
Neuropsychological assessment of patients with BPH/LUTS-ED and controls. (**A**). Battery of 5 psychometric tests: DST, Digit Symbol Test; NCT-A, NCT-B, Number connection tests A and B, respectively; SD, Serial Dotting test, and LTT, Line Tracing test. Scores for the tests are: DST, number of correct pairings; NCT-A, B and SD, time (in seconds) to perform the tests; LTT, time (in seconds) to perform the test plus number of errors. (**B**) Stroop test of attention. Congruent task: number of words read in 45 seconds; Neutral task: number of colors read in 45 seconds; Incongruent task: number of items completed in 45 seconds. (**C**), controls; 0, 3, 6, patients with BPH/LUTS-ED before (0) and after three (3) and six (6) months of tadalafil treatment. Values are expressed as the mean ± SEM. Values significantly different from controls are indicated by asterisks: *p < 0.05; Values significantly different after and before tadalafil treatment are indicated by ^α^p < 0.05; ^αα^p < 0.01; ^ααα^p < 0.001. Values significantly different between 3 and 6 months of treatment are indicated by ^β^p < 0.05.

Neuropsychological assessment. Patients with BPH/LUTS-ED got lower scores (35 ± 3 correct pairings) than controls (42 ± 2 correct pairings) in DST test (p < 0.05) (Fig. 1A), that evaluates mental processing speed. These scores were significantly better (p < 0.001) after 6 months of tadalafil treatment. For NCT-B and LTT tests, which evaluate attention and visuo-spatial coordination, respectively, patients with BPH/LUTS-ED took more time than controls in performing these tasks (for NCT-B: 101 ± 14 vs 70 ± 5 seconds; p < 0.05, and for LTT: 112 ± 9 vs 94 ± 3; p < 0.05) (Fig. 1A). Scores in these tests recovered to control levels after tadalafil treatment (p < 0.05).

Selective attention was assessed with the Stroop test. Controls read 118 ± 2 words in 45 seconds, in the congruent task, whereas patients read fewer words (105 ± 6; p < 0.05) (Fig. 1B). After tadalafil treatment there is a trend to increase this score in the patients, reaching the control values.

In the neutral task, the controls named 82 ± 2 colors; the patients named 74 ± 2 colors, which was significantly lower than controls (p < 0.05), and they recovered to levels similar to controls after 3 months of tadalafil treatment (83 ± 3; p < 0.01).

In the incongruent task patients with BPH/LUTS-ED got lower mean values than controls (45 ± 1 and 49 ± 1, respectively; p < 0.05) (Fig. 1B). There are no changes after tadalafil treatment.

### Inflammation and cGMP

**Figure 2:**
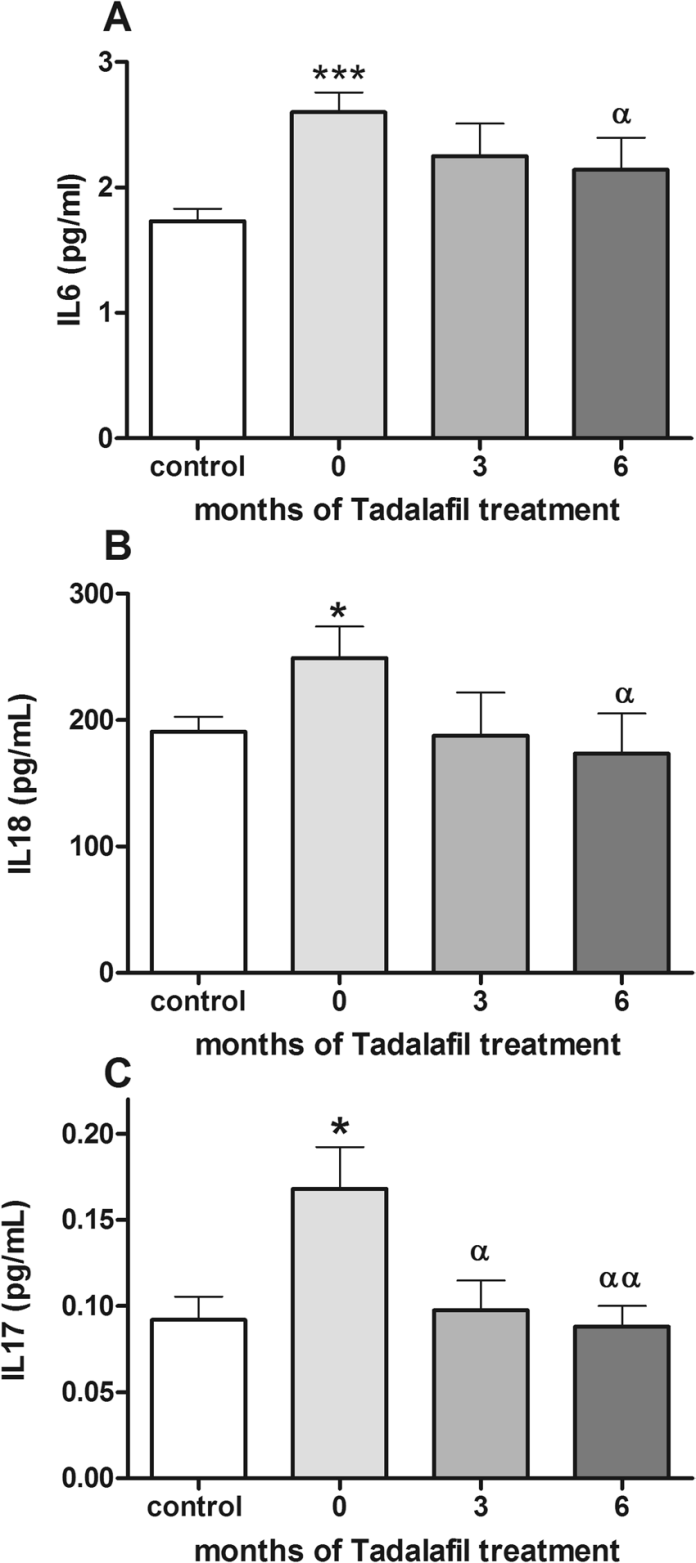
Patients with BPH/LUTS-ED show higher levels of IL-6, IL-18 and IL17 which are reduced after tadalafil treatment. (**A**) Levels of serum IL-6 are increased in patients BPH/LUTS-ED, and are reduced after tadalafil treatment. (**B**) Serum IL-18 levels decreased in patients after tadalafil treatment. (**C**) Levels of serum IL-17 are increased in patients BPH/LUTS-ED before treatment, and are reduced after tadalafil treatment. 0, 3, 6, patients with BPH/LUTS-ED before (0) and after three (3) and six (6) months of tadalafil treatment. Values are expressed as the mean ± SEM. Values significantly different from controls are indicated by asterisks: *p < 0.05; ***p < 0.001. Values significantly different after and before tadalafil treatment are indicated by ^α^p < 0.05; ^αα^p < 0.01.

Figure 2 shows that serum levels of pro-inflammatory interleukins IL-6, IL-18, and IL-17 were significantly increased in patients compared to controls (p < 0.001, p < 0.05, and p < 0.05, respectively). Tadalafil treatment reduced significantly (p < 0.05) these levels after 6 months of treatment, getting values similar to controls.

Regarding plasma cGMP, patients with BPH/LUTS-ED and controls showed similar levels (4.51 ± 0.3, and 4.3 ± 0.3 pmol/ml, respectively). Tadalafil treatment did not change plasma cGMP levels after three (4.55 ± 0.4 pmol/ml) and six months (4.47 ± 0.2 pmol/ml) of treatment.

### Autoreactive T CD4^+^ lymphocytes

**Figure 3:**
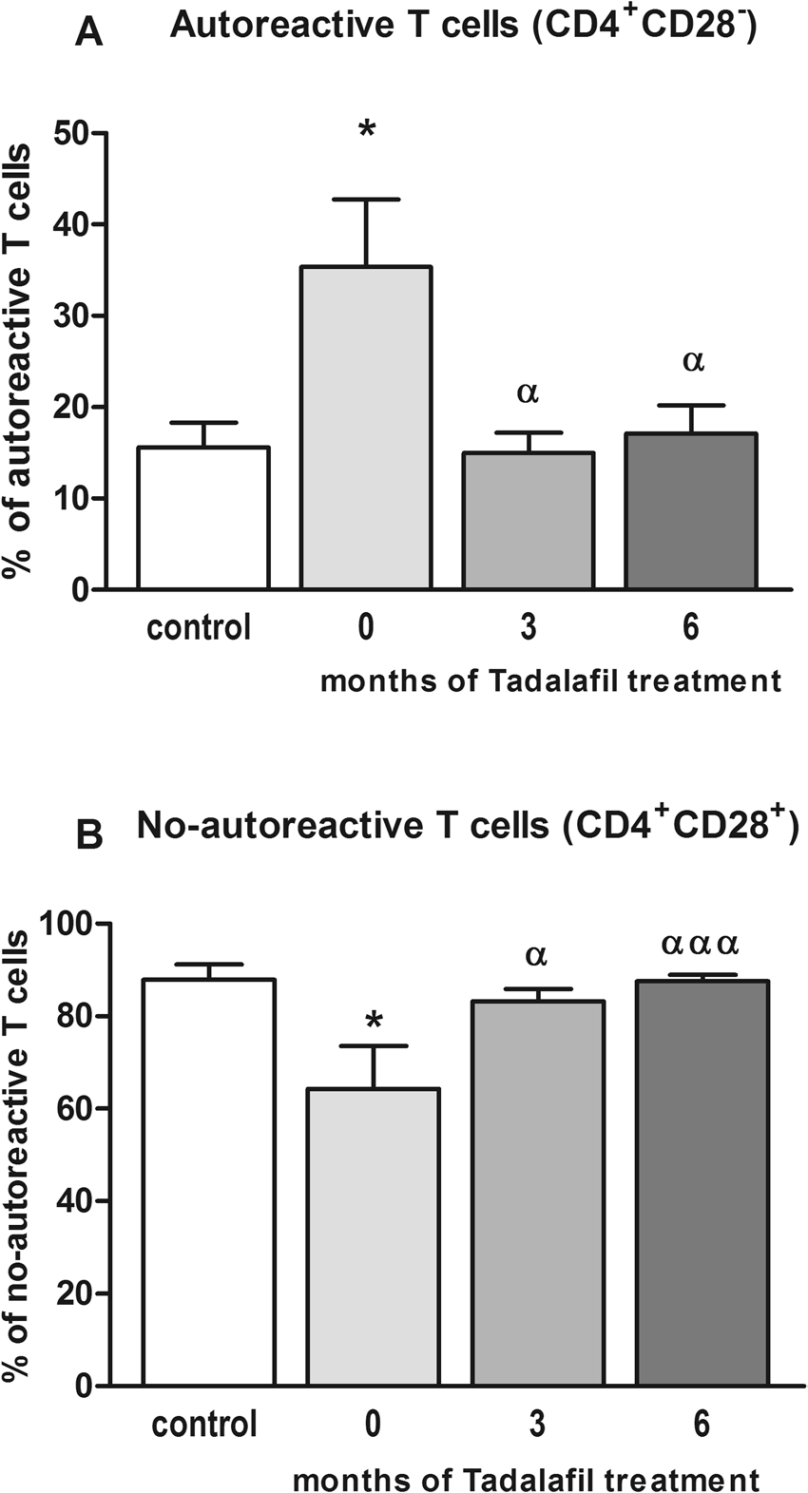
Percentages of autoreactive and no-autoreactive (CD4^+^CD28^+^) T lymphocytes in controls and patients with BPH/LUTS-ED before (0) and after three (3) and six (6) months of tadalafil treatment. (**A**) The percentage of autoreactive (CD4^+^CD28^−^) T lymphocytes is increased in BPH/LUTS-ED patients compared to controls and are normalized after tadalafil treatment. (**B**) The percentage of no-autoreactive T lymphocytes (CD4^+^CD28^+^) is reduced in BPH/LUTS-ED before treatment compared to controls, and increases to control levels after 3 and 6 months of tadalafil treatment. Values are expressed as the mean ± SEM. Values significantly different from controls are indicated by asterisks: *p < 0.05. Values significantly different after and before tadalafil treatment are indicated by ^α^p < 0.05; ^ααα^p < 0.001.

Most CD4^+^ lymphocytes have receptors for CD28, and become activated when they are exposed to CD28. However, there are autoreactive CD4^+^ lymphocytes, which lack the receptor for CD28 (CD4^+^CD28^−^)^16^. Patients with BPH/LUTS-ED showed an increased proportion of CD4^+^CD28^−^ lymphocytes (35 ± 7%) compared to controls (16 ± 3%) (p < 0.05) (Fig. 3A). This proportion was reduced to nearly control levels after three (15 ± 2%; p < 0.05) and six months (17 ± 3%; p < 0.05) of tadalafil treatment (Fig. 3A).

The percentage of no-autoreactive (CD4^+^CD28^+^) T lymphocytes was reduced in parallel with the increase in autoreactive T cells (p < 0.05) (Fig. 3B), to 64 ± 9% in patients *vs*. 88 ± 3% in controls. There was a significantly increase in the proportion of this T lymphocyte population after three (83 ± 3%; p < 0.05) and six months (87 ± 1%; p < 0.001) of tadalafil treatment (Fig. 3B).

### Mismatch negativity (MMN) results

**Figure 4:**
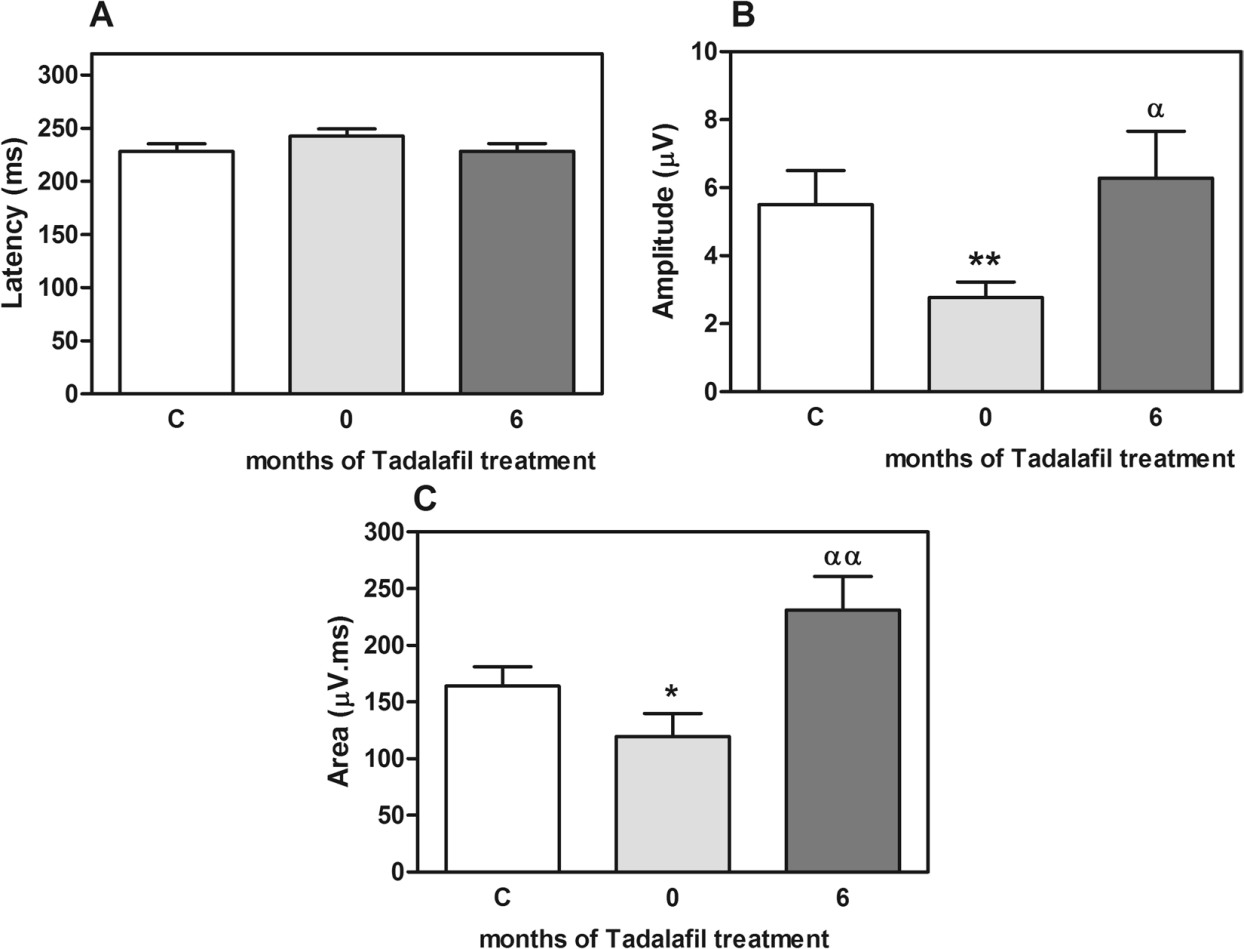
Parameters of mismatch negativity wave (MMN) in controls and patients with BPH/LUTS-ED treated with tadalafil for 6 months. (**A**) MMN Latency. (**B**) MMN Amplitude is reduced in patients compared to controls, and is normalized after tadalafil treatment. (**C**) BPH/LUTS-ED patients showed a lower MMN area than controls that was significantly increased after tadalafil treatment. Values are expressed as the mean ± SEM. Values significantly different from controls are indicated by asterisks: *p < 0.05; **p < 0.01. Values significantly different after and before tadalafil treatment are indicated by ^αα^p < 0.01.

Figure 4 shows the results obtained for the MMN parameters in controls and patients with BPH/LUTS-ED before and after tadalafil treatment. Patients with BPH/LUTS-ED showed a trend to a higher MMN latency (242 ± 7 *vs*. 228 ± 7 ms for controls) (Fig. 4A) and a significantly lower amplitude (1.6 ± 0.4 µV) than controls (5.5 ± 1 µV) (p < 0.01) (Fig. 4B). After 6 months of tadalafil treatment, latency was reduced to 228 ± 7 ms and amplitude was normalized (6.3 ± 1.4 µV) (p < 0.05 *vs*. values before treatment).

**Figure 5:**
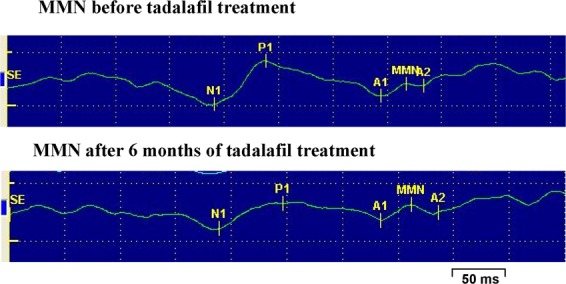
Representative mismatch negativity wave (MMN) obtained before and after treatment with tadalafil in one of the patients with BPH/LUTS-ED. The MMN was calculated by subtracting the event-related potential (ERP) of the standard-stimulus from the deviant-stimulus ERP.

Mean values for MMN area were lower in patients (120 ± 20 µV.ms) than controls (164 ± 17 µV.ms) (p < 0.05), and was significantly increased after six months of tadalafil treatment (231 ± 30 µV.ms) compared to values before treatment (p < 0.01) (Fig. 4C). Figure 5 shows an example of MMN wave obtained before and after treatment with Tadalafil in one of the patients with BPH/LUTS-ED.

### Effects of tadalafil treatment on the IPSS and IIEF5 scores. Correlations with neuropsychological and neurophysiological parameters

There was an improvement of IPSS and IIEF5 scores after 6 months of tadalafil treatment. The mean IPSS score before treatment was of 11 ± 1, that was decreased after treatment (7 ± 1; p = 0.013). The mean score for IIEF5 was 14 ± 1 before treatment, and increased to 21 ± 1 (p = 0.0004) after 6 months of tadalafil treatment. Moreover, there was a good correlation between these scores (IPSS vs. IEEF5: r = −0.618; p = 0.01).

The improvement of IPSS score correlated with an improvement in PHES, NCTA and NCTB scores (r = −0.701, p = 0.002; r = −0.646, p = 0.005; and r = −0.741, p = 0.001, respectively). There was also a trend to be significant correlations between IPSS and DST and LTT tests (r = −0.457, p = 0.07; and r = −0.448, p = 0.07, respectively).

The improvement of IPSS and IIEF5 scores also correlate with amplitude of MMN wave (r = −0.639; p = 0.04, and r = 0.628; p = 0.05, respectively) and there were almost significant correlations of IPSS with the other electrophysiological parameters (latency: r = 0.546, p = 0.06; and area: r = −0.368, p = 0.06).

### Correlation between the psychological and electrophysiological changes with those observed in the inflammatory markers

A decrease in inflammation, measured by IL6 levels, is accompanied by an improvement in the NCTA and NCTB subtests from PHES battery (which evaluate attention) and in visuomotor coordination test, as show the correlations of IL6 levels with these tests (with NCTA: r = −0.403, p = 0.03; NCTB: r = −0.327, p = 0.02; visuomotor: r = −0.443, p = 0.001). There are also significant correlations between IL17 levels and NCTA (r = −0.324; p = 0.02) and Serial Dotting (SDT) test (r = −0.406; p = 0.04).

Moreover, a decrease in the levels of autoreactive (CD4 + CD28^−^) T-lymphocytes and an increase in non-autoreactive (CD4 + CD28 + ) T-lymphocytes correlate with an improvement in attention, measured by the NCTA test (r = −0.330; p = 0.04, and r = 0.690; p = 0.003, respectively).

Regarding the electrophysiological parameters, a decrease in MMN latency correlates with an increase in the number of non-autoreactive (CD4 + CD28 + ) T-lymphocytes (r = −0.757; p = 0.03).

## Discussion

By using an approach combining neuropsychological tests, electrophysiological measures, and biochemical analysis, we show that tadalafil improves the psychometric and attention tests in patients with BPH/LUTS-ED, and that parameters of the “mismatch negativity” event-related brain potential were also normalized after tadalafil treatment. Moreover, tadalafil reduces the peripheral pro-inflammatory interleukins and normalize the subset of autoreactive T cells in patients with BPH/LUTS-ED.

Patients with BPH/LUTS-ED in this study showed some alterations in mental processing speed and attention compared to controls, and after tadalafil treatment the scores in psychometric tests improved. MMN area improved in parallel with performance in mental processing speed and attention, as measured by DST test, given the good correlation found (r = 0.665; p = 0.018).

To the best of our knowledge this is the first study on effects of tadalafil on event-related brain potentials. MMN is an auditory event-related cognitive potential that reflects an attentional trigger. Multiple neuronal elements generate the MMN wave. MMN latency depends on the response speed of the neurons, whereas the amplitude is related to the maximum response of the sum of all the neurons that respond synchronously. The area is related to the accumulated response of all neurons during the entire MMN wave until returning to basal levels. In patients with BPH/LUTS-ED there are no alterations in latency, but the amplitude and the area are reduced, which is indicative of the activation of a lower number of neurons and during shorter periods. Tadalafil treatment normalizes these parameters, indicating an improvement of cortical activity.

This study agrees with those from Shim *et al*.^14^, who showed that a daily dosing with udenafil for 2 months improved cognitive function, in addition to erectile function, in patients with ED. Tadalafil has been reported to improve cognitive function in a mouse model of Alzheimer’s disease^17^.

A study assessing the central side-effects of sildenafil on attention and memory functions in healthy young men^18^ reported higher amplitude of P3 component of event-related potential (ERP) suggesting a more availability of processing resources in the sildenafil condition, and an increased capacity to focus attention on streams of auditory stimuli. Our results indicate that tadalafil would exert similar effects in patients with BPH/LUTS-ED.

The mechanisms by which tadalafil improves cognition in patients with BPH/LUTS-ED could be related with the function of tadalafil as PDE5 inhibitor. PDE5 is specific for cGMP hydrolysis, and enhances cGMP signaling via reducing the degradation of this cyclic nucleotide^19^. Patients in this study were treated with a daily low-dose of tadalafil (5 mg/day), which did not increase the amount of plasma cGMP, but had positive effects on cognitive functions in patients, given the improvement in psychometric tests and MMN parameters. As tadalafil has been reported to cross the blood-brain barrier^17^, it may affect attention-related processes by promoting increases of cGMP in cortical neurons. Compounds that restore pathway function and cGMP levels restore learning capacity in rats^7^.

BPH is associated with inflammation^8,9^ and with chronic activation of prostate-infiltrating lymphocytes^20^. In this study we show that patients with BPH/LUTS-ED present higher levels of pro-inflammatory interleukins in peripheral blood. Moreover, a subset of autoreactive T lymphocytes (CD4^+^ CD28^−^) are increased in blood from these patients (about two-fold compared to controls), indicating a pro-inflammatory environment in their blood which would be affecting in some degree to cognitive functions such as attention. CD4^+^ CD28^−^ T cells expand in several diseases associated with chronic inflammation (e.g. autoimmunity, atherosclerosis, etc)^16^.The increased amount of CD4^+^ CD28^−^ T cells in patients with BPH/LUTS-ED, which can secrete inflammatory cytokines such as interferon-γ (IFN-γ) and tumor necrosis factor-α (TNF-α), could be promoting the differentiation of CD4^+^ T cells to IL-17 producers, process in which TGF-β and IL-6 are involved, and that is amplified by TNF-α and IL-1β^21^. Moreover, dysregulation of the immune response in patients with BPH/LUTS-ED can be through the high expression of IL-17 that stimulates the production of IL-6, which contributes to stromal growth in BPH^9^. In our study, patients with BPH/LUTS-ED showed increased levels of pro-inflammatory cytokines IL-6, and IL-17. IL-17 is an important player in the establishment of prostatic inflammation, and can be considered a modulator of BPH immune responses, by maintaining the local inflammatory microenvironment and amplifying the damage to prostatic tissue^8^.

In patients with BPH/LUTS-ED, PDE5 inhibition by tadalafil treatment exerts an anti-inflammatory effect by normalizing the levels of these proinflammatory interleukins and the levels of autoreactive T lymphocytes. This result agrees with Vignozzi *et al*.^22^, who demonstrated that tadalafil possesses antioxidant as well as antinflammatory action in addition to its vasodilatory property, and that PDE5 inhibitors suppressed other TNFα-induced genes related to inflammation (IL-6, MCP1, IL-12, COX2). The changes observed in peripheral blood could reflect those in prostatic tissue.

Moreover, this anti-inflammatory effect of tadalafil is associated to an improvement in attention and coordination functions, given the correlations found between the psychometric tests and proinflammatory interleukins, and with levels of autoreactive T-cells, as well as with IPSS scores.

We also found higher serum IL-18 levels in patients with BPH/LUTS-ED than controls. This interleukin was found to be expressed at significantly higher levels in BPH tissues, both rat and human, than in normal prostate tissues, and may act directly in BPH pathogenesis by inducing Thrombospondin-1 production in prostatic smooth muscle cells via Akt phosphorylation^23^.

## Conclusion

In conclusion, there is a pro-inflammatory environment in blood from patients with BPH/LUTS-ED which would be affecting in some degree to cognitive functions and auditory evoked potential. PDE5 inhibition by tadalafil exerts an anti-inflammatory activity, and ameliorates psychometric tests, and MMN parameters. As tadalafil requires less frequent administration than other PDE5 inhibitors and it is safe in chronic treatments it may be a promising candidate for chronic treatment in other inflammatory pathologies associated with mild cognitive impairment.

## Patients and Methods

Participants. Nine patients with BPH/LUTS-ED (50–70 years old) were enrolled after written informed consent. Patients were diagnosed for BPH/LUTS by the International Prostate Symptom Score (IPSS), getting a score from low to moderate (mean IPSS score: 11 ± 1; ranging from 9 to 18). Patients included presented a mild to moderate erectile dysfunction, with a mean score for IIEF5 (International Index of Erectile Function) of 14 ± 1, ranging from 12 to 17. At time of entering the study, the patients did not take any medication. Mean values for analytical parameters were: mean PSA levels: 1.26 ± 0.3 ng/mL (normal range 0–4 ng/mL); mean Testosterone levels: 495 ± 51 ng/dL (normal range 241–827 ng/dL). Exclusion criteria were: high PSA levels (>4 ng/mL); a rectal examination suspicious for prostate cancer, other diagnosed pathology; taking any other medication. Other urological pathologies (vesical tumours, litiasis…) were discarded by abdominal echography.

Twelve age-matched healthy controls (mean age: 62; range: 50–70) without BPH/LUTS-ED were also included, after written informed consent. All subjects were volunteers.

Psychometric tests, attention Stroop test and blood extraction were performed on the same day. The auditory evoked EEG potential, “mismatch negativity” (MMN), was performed in the following week after psychometric tests.

Patients with BPH/LUTS-ED were treated by the urologist with 5 mg/day of tadalafil (Cialis^®^, Eli Lilly Nederland B.V.). Neuropsychological assessment and blood extraction were performed at 3 and 6 months of tadalafil treatment; MMN study was repeated after 6 months of treatment. Study protocols were approved by Scientific and Ethical Committee of Arnau de Vilanova hospital, Valencia, Spain, and conform to ethical guidelines of Helsinki Declaration.

### Neuropsychological assessment

We used a battery of five psychometric tests (PHES, Psychometric Hepatic Encephalopathy Score) which is usually used for the diagnosis in other pathologies with mild neurological impairment such as in minimal hepatic encephalopathy^24^.This battery is composed of five psychometric tests: Digit symbol test (DST), number connection test A and B (NCT-A and NCT-B), serial dotting test (SDT), and line tracing test (LTT). Each subtest from PHES battery assesses different cognitive domains: DST: processing speed and working memory; NCT-A and NCT-B: mental processing speed and attention; SDT and LTT: visuo-spatial coordination.

A color-word version of Stroop test was used for assessing selective attention. Controls and patients performed sequentially the congruent, neutral and incongruent tasks, 45 seconds per task, as in Felipo *et al*.^15^.

### Determinations of Interleukins and cyclic GMP

Interleukins IL-6, IL-18, and IL-17 were measured in serum using Human IL-6 and IL-18 Platinum ELISA kits, and Human IL17 high sensitivity ELISA kit, respectively, from eBioscience (Bender MedSystems GmbH, Vienna, Austria). Cyclic GMP (cGMP) in plasma was determined using the BIOTRAK cGMP enzyme immunoassay kit from Amersham (GE Healthcare, Life Sciences, Buckinghamshire, UK).

### Flow cytometry analysis

Flow cytometry analysis was performed as in Mangas-Losada *et al*.^25^. Fifty μL of whole blood was incubated with a mixture of monoclonal antibodies specific for the different leukocyte subpopulations (see below) and with 2 mL BD FACS Lysing Solution 1 × (Becton, Dickinson and Company, Franklin Lakes, NJ, USA). Samples were incubated in the dark for 10 minutes at room temperature. Then, 50 µl of Flow Count (Beckman Coulter, Miami, FL, USA) was added to quantify the number of cells per microliter. Analysis was performed on a Gallios flow cytometer (Beckman Coulter, Miami, FL, USA) and data were analyzed with the Kaluza software package.

### Monoclonal antibodies used

Different cell populations were labelled with antibodies to CD45 (total leukocytes), CD3 (T lymphocytes), CD4 (T helper lymphocytes), CD28 (negative selection for autoreactive T helper lymphocytes). The antibodies used were the following: CD45-Krome Orange (clone J.33) (CD45-KO), and CD4-PhycoerythrinTexas Red-X (Clone SFCI12T4D11 (T4)) (CD4- ECD), from Beckman Coulter (Miami, FL, USA); CD3- Allophycocyanin (clone UCHT1) (CD3-APC), and CD28-Pacific Blue (clone CD28.2) (CD28-PB) from Biolegend (San Diego, CA, USA).

### Mismatch negativity study

Controls and patients performed the mismatch negativity (MMN) analysis as previously described^15^ using an 8-channels system for evoked potentials (NeuropackM1, Nihon-Kohden) and software adapted for MMN. The stimulation protocol used a stimulus train consisting of a sequence of standard tones of one frequency and duration (10 ms) which were followed by an inter-train interval of 300 ms. The first tone of the next train (differing in frequency) correspond to the “deviant”. Twelve frequencies ranging in 50 Hz steps from 750 to 1250 Hz were used. The number of tones in each stimulus train varied randomly and can be 2, 4, 8, 16 or 36. A total of 4500 stimuli and 400 deviants were delivered. During the 45 min EEG recording, subjects watched a silent self-selected video film. EEG was recorded continuously from electrodes Fz, F3, F4, Cz, left and right mastoids placed according to the international 10–20 system. The vertical electrooculogram was recorded from electrodes placed above the right eye and the right outer canthus. System band pass was 0–70 Hz, with a digital sampling rate of 500 Hz. The ground electrode was placed on the central forehead and reference on the bridge of the nose. Data were analyzed as in Felipo *et al*.^15^ The latency (ms), amplitude (μV) and area (μV. ms) of the resulting MMN wave were calculated. In patients treated with tadalafil, the MMN was analyzed twice, before and 6 months after treatment with tadalafil to assess the evolution.

### Statistical analysis

All data are expressed as mean values ± standard error (SEM). The Kolmogorov-Smirnov test was used for testing whether the variables had a normal distribution.

Differences between controls and patients before treatment were analyzed by t-student test or Mann Whitney test (for non-parametric variables). Differences between before and after 3 and 6 months of tadalafil treatment were analyzed by repeated measures ANOVA followed by post-hoc Tukey’s multiple comparison test, except for non-parametric variables in which Friedman test followed by Dunn’s test was used. As mismatch negativity was carried out before and after 6 months of treatment, results were analyzed using a Paired *t*-test, except for non-parametric variables, in which Wilcoxon matched-pairs signed rank test was used. The significance level was set at P < 0.05. The statistical analyses were performed using the Graphpad Prism 6.0. Pearson correlation analyses were performed with SPSS (Version 22.0, Chicago, IL).

## Data Availability

All data generated or analysed during this study are included in this published article.
